# Holistic engineering of Cal-A lipase chain-length selectivity identifies triglyceride binding hot-spot

**DOI:** 10.1371/journal.pone.0210100

**Published:** 2019-01-14

**Authors:** Daniela Quaglia, Lorea Alejaldre, Sara Ouadhi, Olivier Rousseau, Joelle N. Pelletier

**Affiliations:** 1 Département de Chimie and Center for Green Chemistry and Catalysis (CGCC), Université de Montréal, Montréal, QC, Canada; 2 PROTEO, The Québec Network for Research on Protein Function, Engineering and Applications, Québec, QC, Canada; 3 Département de Biochimie, Université de Montréal, Montréal, QC, Canada; University of Tennessee, UNITED STATES

## Abstract

Through the application of a region-focused saturation mutagenesis and randomization approach, protein engineering of the Cal-A enzyme was undertaken with the goal of conferring new triglyceride selectivity. Little is known about the mode of triglyceride binding to Cal-A. Engineering Cal-A thus requires a systemic approach. Targeted and randomized Cal-A libraries were created, recombined using the Golden Gate approach and screened to detect variants able to discriminate between long-chain (olive oil) and short-chain (tributyrin) triglyceride substrates using a high-throughput *in vivo* method to visualize hydrolytic activity. Discriminative variants were analyzed using an in-house script to identify predominant substitutions. This approach allowed identification of variants that exhibit strong discrimination for the hydrolysis of short-chain triglycerides and others that discriminate towards hydrolysis of long-chain triglycerides. A clear pattern emerged from the discriminative variants, identifying the 217–245 helix-loop-helix motif as being a hot-spot for triglyceride recognition. This was the consequence of introducing the entire mutational load in selected regions, without putting a strain on distal parts of the protein. Our results improve our understanding of the Cal-A lipase mode of action and selectivity. This holistic perspective to protein engineering, where parts of the gene are individually mutated and the impact evaluated in the context of the whole protein, can be applied to any protein scaffold.

## Introduction

Enzyme engineering is widely used to transform enzymes into better catalysts for improved specificity, selectivity, stability, and new chemistry [1–4]. Methods available to produce the genetic diversity necessary to discover variants with improved characteristics range from focussed point mutations to a completely randomized approach. The diversified library of variants is then screened for the desired properties [5–9]. Many of these advances attempt to address two critical limitations to efficient enzyme engineering: the need to reduce library sizes by pinpointing mutational hot-spots where engineering will have the most effect, and the unavailability of effective high-throughput screening methods that directly elicit the desired property, rather than a proxy to that property. It is of paramount importance for advancement in the area to develop better approaches for the creation of smart libraries of mutants [10, 11] and tools for direct screening.

Smart library creation is currently best addressed either by performing series of sequential and converging mutagenesis campaigns on the whole gene of interest, or by inclusion of computational predictions based on structure and sequence alignments [5, 6, 8, 9, 12–15]. We tackle the problem from a dynamic and more holistic perspective, applying region-focused saturation mutagenesis and randomization with seamless recombination [11]. In this approach, individual regions of the gene of interest are treated for mutagenesis and are readily recombined with other mutated or native regions. This enables customized generation of mutations in each region, followed by recombination into the full-length coding sequence through assembly using the Golden Gate method [11, 16–18]. The flexibility of the method resides in the ability of introducing different patterns of mutations in distinct regions of the protein: each part can be mutated randomly or using a more targeted approach such as saturation mutagenesis.

Demonstrated at a small scale in previous work [11], here we validate this flexible, combinatorial approach by expanding the engineered libraries. Then, we demonstrate that this approach readily defines mutational hot-spots in the chosen target protein (*Candida antarctica Lipase A*, Cal-A). We rapidly discovered improved enzyme variants that were not restricted to point mutants but considered possible synergistic effects between substitutions [19]. We further demonstrated that the variants allowed individuation of point mutants of interest, providing an important body of information on the target enzyme, Cal-A.

Growing industrial interest in the development of biocatalysts for selective hydrolysis of fatty acids from naturally-sourced triglycerides has stimulated research and development concerning lipases. Cal-A has been investigated for a range of biocatalytic applications, such as using the native Cal-A to concentrate ω-3 polyunsaturated fatty acids (PUFA) from marine resources [20, 21] or engineering variants for the selective hydrolysis of *trans*-fatty acids [22, 23]. Lipases with the ability to hydrolyze fatty acids of specific chain-lengths are also highly desirable, particularly for the food industry [24, 25]. For instance, medium-chain fatty acids constitute high-value food additives as they provide quick access to energy. Furthermore, health benefits have been associated with milk-fat products rich in diglycerides composed of short-chain saturated fatty acids [24–26].

Cal-A is thermostable (> 90°C) at acidic pH, it accepts a wide variety of substrates including sterically hindered ones. Furthermore, it differs from the majority of lipases in that it does not undergo extensive movement of a lid domain when undergoing interfacial activation [22, 27, 28]. Interfacial activation is a fundamental property of most lipases, where association with lipids is required for a large conformational change that provides access to the otherwise masked active site. These qualities reduce the complexity of the system and make Cal-A a good target for engineering new properties. Prior reports interpreted a co-crystallized PEG-4 molecule as being a fortuitous substrate surrogate, which allowed to identify a docking region for *p*NO_2_-phenyl derivatives of fatty acids. This was supported by the demonstration of altered chain-length selectivity upon mutating that region [29]. Specifically, mutations Gly237Lys/Val/Tyr and Val290Trp near the PEG-4 procured enhanced hydrolysis of medium-chain (C6-C10) and long-chain (C22) fatty acid substrates [22, 23, 30], demonstrating the potential for chain-length alteration in Cal-A.

In this work, we engineered Cal-A using the region-focused, random and targeted mutagenesis approach to enhance its discriminative capability to hydrolyze short- (tributyrin, C-4) *vs* long-chain (olive oil, approximately 80% C-18 and 20% C-16) fatty acid esters [31], obtaining variants displaying either selectivity. Identification of variants with the desired selectivities was made possible by applying a high-throughput, whole-cell visual screen on solid media containing tributyrin or olive oil [11]. A particular emphasis was placed on mutations in the PEG-4-binding region [29]. Finally, we developed a straightforward script to visualize the sites of mutation associated with either discriminative phenotype. By this computational approach, a large body of mutational data was readily analyzed and clearly revealed the main hot-spot for modulation of substrate binding. Quantitative analysis of activity was achieved for some of the variants with a short- and long-chained *p*NO_2_-phenyl esters substrates, the industry standard for monitoring lipase activity. This confirms that variants with a markedly higher activity for both triglycerides and *p*-NO_2_-phenyl derivatives of short- or long- chain fatty acids were identified.

## Results and discussion

### Library generation and characterization

**Fig 1:**
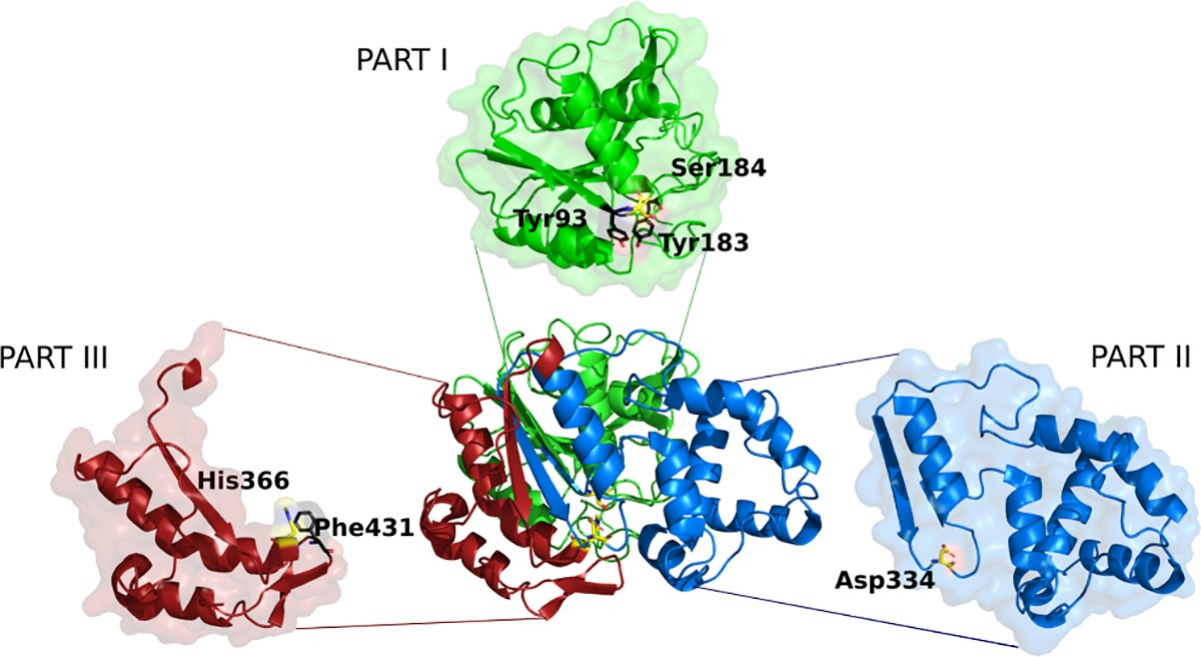
Cal-A was treated in three segments for mutagenesis. Parts 1 (encoding the *N*-terminal 210 residues), 2 (encoding residues 211 to 350) and 3 (*C*-terminal residues 351 to 446 plus 6His-tag) were individually randomized and recombined with wild-type or mutated parts. Parts 1 and 3 include residues treated by NDT saturation mutagenesis (Tyr93, Tyr183 and Phe 431), shown in magenta. The catalytic triad (Ser184, Asp334, His366) is shown in yellow. Image generated using PDB coordinates 2VEO [29].

Cal-A was treated for random mutagenesis in three segments (referred to as ‘parts’) (Fig 1). We previously reported that the separation of Cal-A into three parts had facilitated random mutagenesis of Part 2 (residues 211–350) in isolation from the *N*-terminal Part 1 and *C*-terminal Part 3 [11]. Part 2 includes a putative tunnel, hypothesized to bind the long chain of a fatty acid substrate [22]. Following random mutation, it had been seamlessly recombined with the wild-type Parts 1 and 3 to give ‘Random 2’ library (Table 1) [11]. Recombination was achieved with the Golden Gate-based method that is ideally suited for inserting randomized segments into specific parts of a gene [11, 16, 17]. In separate experiments, residues in Parts 1 and 3 that could potentially alter the discriminative hydrolysis had been individuated according to structural analysis: Tyr93, positioned near the active site, was selected for its potential ability to modulate substrate access; the sterically hindered Tyr183 appeared to open a putative tunnel during molecular dynamics simulations [11], whereas Phe431 is a hypothetical gate keeper, modulating substrate entry [27]. These three positions were mutated using NDT saturation mutagenesis (N = A,C,G,T, and D = A, G or T; covering 12 codons, 12 amino acids) and recombined with the appropriate wild-type or mutated parts. Screening yielded between 10–40% of clones in the various libraries with improved discrimination towards either short- or long-chain fatty acid esters relative to wild-type Cal-A. This validated the method used to create the desired functional diversity, although the identity of variants of interest had not been determined.

**Table 1:** Library generation and assessment of library quality.

Library	Randomization method	Mutations per gene segment (/kb)	Mutations per reassembledgene ^a^	Total bp sequenced in Cal-A coding region
**Random 1** (605 bp)	Randomized Part 1 + WT Parts 2,3	2.1	1.3	11000
**Random 2**^**b**^ (420 bp)	Randomized Part 2 + WT Parts 1,3	4.1	1.7	8220
**Random 3** (286 bp)	Randomized Part 3 + WT Parts 1,2	3.3	1	4480
**Random Rec**	Assembly of randomized Parts 1,2, 3	3.0	3.9	18304
**Random Tot**	Randomization of full coding sequence	4.2	5.5	19155

^a^ Length of the reassembled gene: 1311 bp.

^b^ Random 2 library was previously reported [11]. Additional DNA sequencing was performed in this study.

Encouraged by the promising functional diversity that can be accessed and seeking further improvements in discrimination, we explored the sequence space of Cal-A more widely by applying broader randomization and seamless recombination. We complemented the existing libraries [11] by randomizing Part 1 and Part 3 (Fig 1). Each randomized part was integrated into the native gene, yielding libraries ‘Random 1’ and ‘Random 3’, respectively (Table 1) [11]. The randomly mutated parts 1, 2 and 3 were also recombined together, yielding a reassembled, fully randomized Cal-A library (library ‘Random Rec’).

Briefly, the parts of Cal-A were synthesized by ATUM (formerly DNA2.0) and delivered in plasmid pM269; these part-carrying constructs are refered to as ‘mother vectors’. Reassembly of the parts using the Golden Gate strategy (through the use of *Bsa*I and S*ap*I type IIS restriction enzymes) was carried out in an Electra expression vector, refered to as a ‘daughter vector’ (pD441pelB, DNA2.0), which is under the control of T5 promoter and carries a pelB leader sequence for periplasmatic expression, as previously described [11]. For randomization, the parts were subjected to error-prone mutagenesis in the mother vectors and reassembled in the daughter vector with the complementary native parts, to obtain libraries Random 1 and Random 3. The randomized Parts 1 and 3 were also assembled with Random 2 (previously obtained) in the daughter vector to give the fully randomized Random Rec. In parallel, we also randomized the entire gene as a single entity (not in parts; library ‘Random Tot’, Table 1) to compare the efficiency of randomizing and reassembling separate parts with the complete randomization of the gene. DNA sequencing confirmed the quality of the newly obtained libraries (Table 1).

The number of mutations obtained per individually mutated part (hence per gene, when reassembled with native parts) during randomization of Parts 1 and 3 (libraries Random 1 and 3, respectively) is comparable: an average of 1.3 and 1 mutation per gene (Table 1). This mutation rate is similar to that previously obtained for randomization of Part 2 (library Random 2, 1.7 mutations per gene) [11]. Libraries Random Rec and Random Tot have a greater mutational load because the entire length of the gene is randomized (either by full gene randomization or by recombination of randomized parts): 5.5 and 3.9 mutations per gene, respectively. It is satisfying to note that the mutational load of the Random Rec library, obtained by recombination of libraries Random 1, 2 and 3, is essentially equal to the sum of the average mutations of each part (1.3 +1.7 +1 = 4.0). This confirms the validity of the combinatorial assembly method as a reliable tool to obtain quality libraries.

### Screening for discriminative hydrolysis of short- or long-chain esters

The randomized libraries were screened towards two classes of substrates: triglycerides and activated *p*NO_2_-phenyl-esters of different lengths. Triglycerides are the natural substrates of Cal-A and their hydrolysis is the ultimate industrial goal. We previously reported a rapid and robust automated method for screening against triglycerides on solid medium plates to procure a high-throughput platform for primary screening of libraries [11]. Here, solid emulsions containing either tributyrin or olive oil show haloes surrounding colonies that hydrolyze that substrate. For tributyrin emulsions, clear haloes are observed; olive oil emulsions require the addition of rhodamine where the change of pH upon hydrolysis gives rise to increased fluorescence. Observation of halo formation on growth medium supplemented with emulsified triglycerides is amenable to high-throughput screening yet provides only a qualitative assessment. Selectivity was determined by comparing activity against individual substrates; we note that this method is incompatible with multi-substrate competition [32, 33]. *p*NO_2_-Phenyl-esters were used as a secondary substrate in this study: despite being an indirect assessment of activity toward triglycerides, they are rapid, quantitative and practical and as thus widely used as standards to assess lipase activity ([34]and references therein). They are single-chain fatty acid esters rather than glycerol triesters, and the *p*NO_2_-substituent activates them toward hydrolysis. Despite differing significantly from native triglycerides in structure and in reactivity, they allow for direct quantitative activity measurements by spectrophotometry and provide a benchmark for comparison with current literature of the reactivity of key variants towards specific fatty acid esters.

We performed automated high-throughput screening on solid media to evaluate the selectivity of Cal-A variant libraries towards the hydrolysis of short-chain triglycerides (tributyrin) and long-chain fatty acid esters (olive oil, which contains ≈ 70% oleic acid in a triglyceride form) [11]. Briefly, rectangular dishes were cast with agar growth media containing either tributyrin or olive oil/rhodamine to form an opaque emulsion. Using a liquid handler, cultures of *E*. *coli* BL21(DE3) carrying individual variants from the Cal-A libraries and propagated in 96 deep-well plates were spotted onto the solid media. The inoculated plates were incubated at 30°C overnight (approximately 16 hrs) to obtain colonies of 0.2–0.5 cm in diameter. The solid emulsions containing either tributyrin or olive oil show haloes surrounding colonies that hydrolyze that substrate. For tributyrin emulsions, clear haloes are observed; olive oil emulsions require the addition of rhodamine where the change of pH upon hydrolysis gives rise to increased fluorescence [11, 35, 36]. Variants of interest were selected based on strong hydrolysis of one substrate and weak or no hydrolysis of the other (Table 2).

**Table 2:** Discriminative variants obtained upon screening the Cal-A libraries against short- and long-chain triglycerides.

Library	Clones screened	Active^a^	Discriminative towards	
			short-chain	long-chain
**Random 1**	384	89%	0	0
**Random 2**	384	66%	18	3
**Random 3**	384	89%	0	0
**Random Rec**	768	41%	12	6
**Random Tot**	768	23%	10	4

^a^ Variants were screened for halo formation around colonies spotted via automation on solid medium. Variants were considered active only when halo formation was unequivocal.

Despite specific variants of Part 1 and Part 3 having been identified as being discriminative in point mutant libraries (refer to previous work [11], and below), none were identified upon randomization of Parts 1 and 3, suggesting that there are few discriminative possibilities in those segments of Cal-A. In contrast, all libraries that include randomization of Part 2 (libraries Random 2, Random Rec and Random Tot) yielded several variants of both discriminative phenotypes. The region defined by Part 2 contains the putative fatty acid-binding tunnel [22]. Interestingly, library Random 2 yielded a higher rate of active variants than Random Rec or Random Tot. This appears to result from having a lower but more focused mutational load. As a result, fewer screening efforts (384 *vs* 768) yielded a greater number of discriminative variants in library Random 2 (21 total) than in libraries Random Rec or Random Tot (18 and 14, respectively). These results clearly illustrate the additional level of control gained by a segment-wise approach to mutagenesis.

These and previous efforts [11] provided us with a diverse pool of variants that were either point-mutated at positions Tyr93 (Part 1), Tyr183 (Part 1), Phe431 (Part 3) or randomized entirely or in part, and exhibited discrimination in hydrolysis of short- or long-chain triglycerides. In addition to the results presented here, we also applied the flexible reassembly strategy to recombine libraries point mutated in Parts 1 and 3 (namely, Try93/Phe431, and Tyr183/Phe431), to uncover potential synergistic effects. No improved discriminative variants were obtained from these combinations and they are not further discussed. All selected variants were characterized by DNA sequencing to gain insight into the acquired discriminative abilities.

#### Characterization of point substitutions procuring discrimination for the hydrolysis of short- or long-chain triglycerides

**Fig 2:**
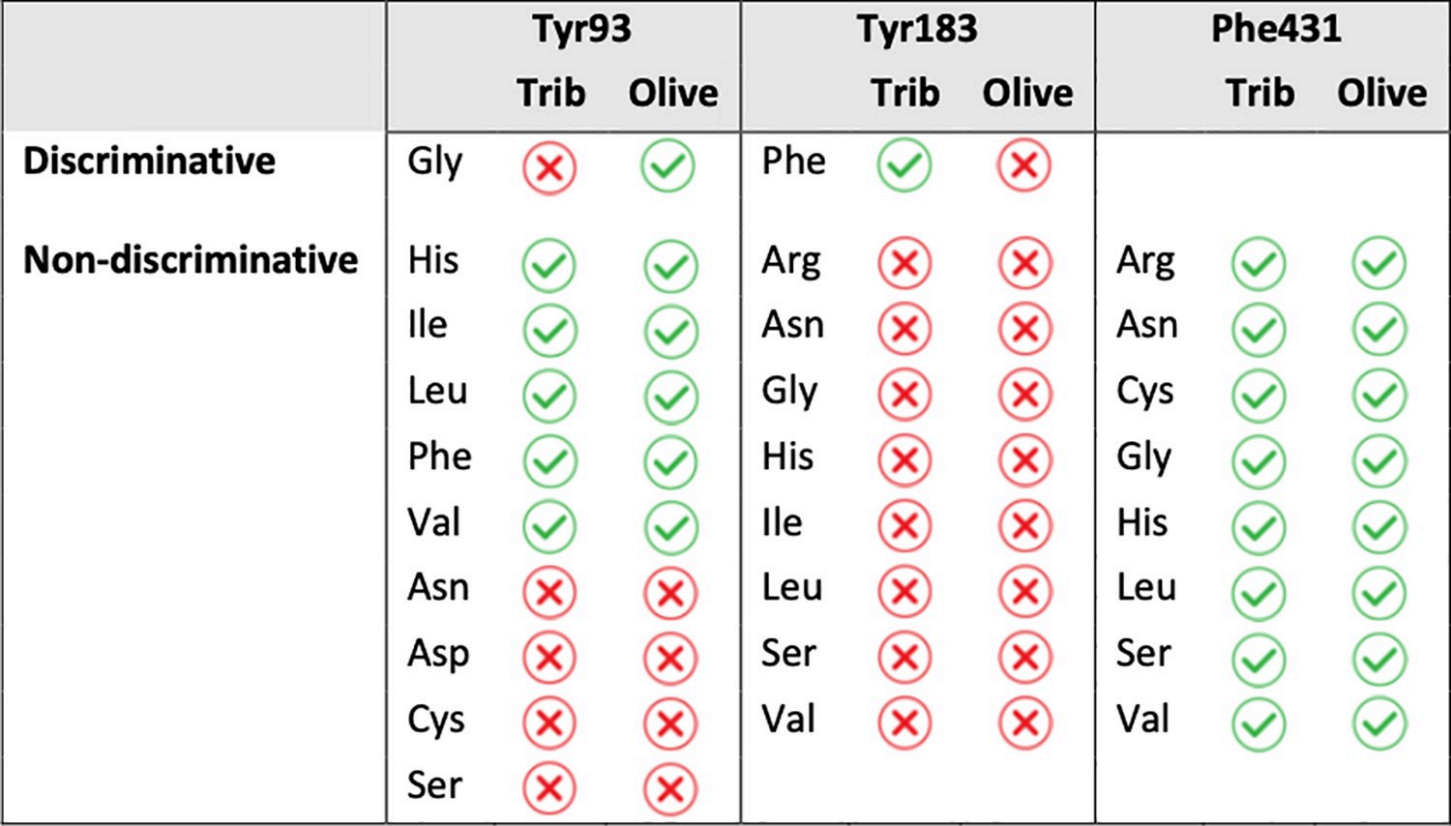
Short- *vs* long-chain hydrolytic activity in point substituted libraries of Tyr93, Tyr183 and Phe431. Variants were screened for halo formation around colonies spotted on solid medium. Hydrolysis of the short-chain tributyrin (Trib) or long-chain olive oil (Olive) were rated as active (green checkmark) or inactive (red ‘Χ’).

When Tyr93 was substituted with Gly, Cal-A lost its ability to process tributyrin while maintaining the ability to process olive oil, making Tyr93Gly discriminative towards hydrolysis of long-chain fatty acids (Fig 2). Substitution of Tyr with small, polar residues (Ser, Cys, Asn and Asp) resulted in loss of activity for both substrates while larger, hydrophobic substitutions (His, Phe, Val, Leu, Ile) were comparable in activity to the wild-type.

Most of the Tyr183 substitutions we identified abolished activity (Gly, Asn, Arg, Ser, His, Ile, Val and Leu). Only Tyr183Phe was active, and it was active only toward tributyrin, suggesting that Tyr183Phe is discriminative for hydrolysis of short-chain triglycerides. In contrast, Phe431 was highly permissive to substitution. Activity was maintained toward long and short-chain triglycerides in all variants obtained: Gly, Asn, Cys, Ser, Arg, His, Val, Leu (Fig 2).

These results inform us of the utility of varying Cal-A positions 93 and 183 (belonging to Part 1) to alter substrate discrimination, while position 431 (belonging to Part 3) was not productively altered toward this goal. In addition to acquiring information on the tolerance of these positions to substitution, it is gratifying that we obtained both discriminative phenotypes with point mutations in a single region (Part 1).

#### Identification of the substitutions in randomized variants procuring discriminative hydrolysis

Analysis of discriminative variants selected from randomly mutated libraries required a different approach because they generally included multiple amino acid substitutions. As a result, their acquired discriminative ability can be due to one or more among these. We turned to a 3D mapping approach to provide a holistic determination of the key regions that confer either discriminative capacity to Cal-A.

A script was created to align each variant sequence with the Cal-A reference sequence and identify amino acid substitutions. Each variant was assigned a phenotype describing either activity towards, or capacity to discriminate between, short- and long-chain triglycerides. The variants, which contain between 1 and 6 mutations, were ranked from inactive to highly active according to halo formation upon triplicate in-plate screening, the wild-type Cal-A serving as a reference activity level (S1–S3 Tables). Each defined phenotype, linked to the corresponding mutations in each variant, was assigned a value that served for 3D visualization on the structure of Cal-A. This approach allowed us to rapidly analyze the randomized variants from the perspectives of activity towards a given substrate and discrimination between substrates, and gain insight into key regions and residues that altered function.

#### Activity levels of discriminative variants

**Fig 3:**
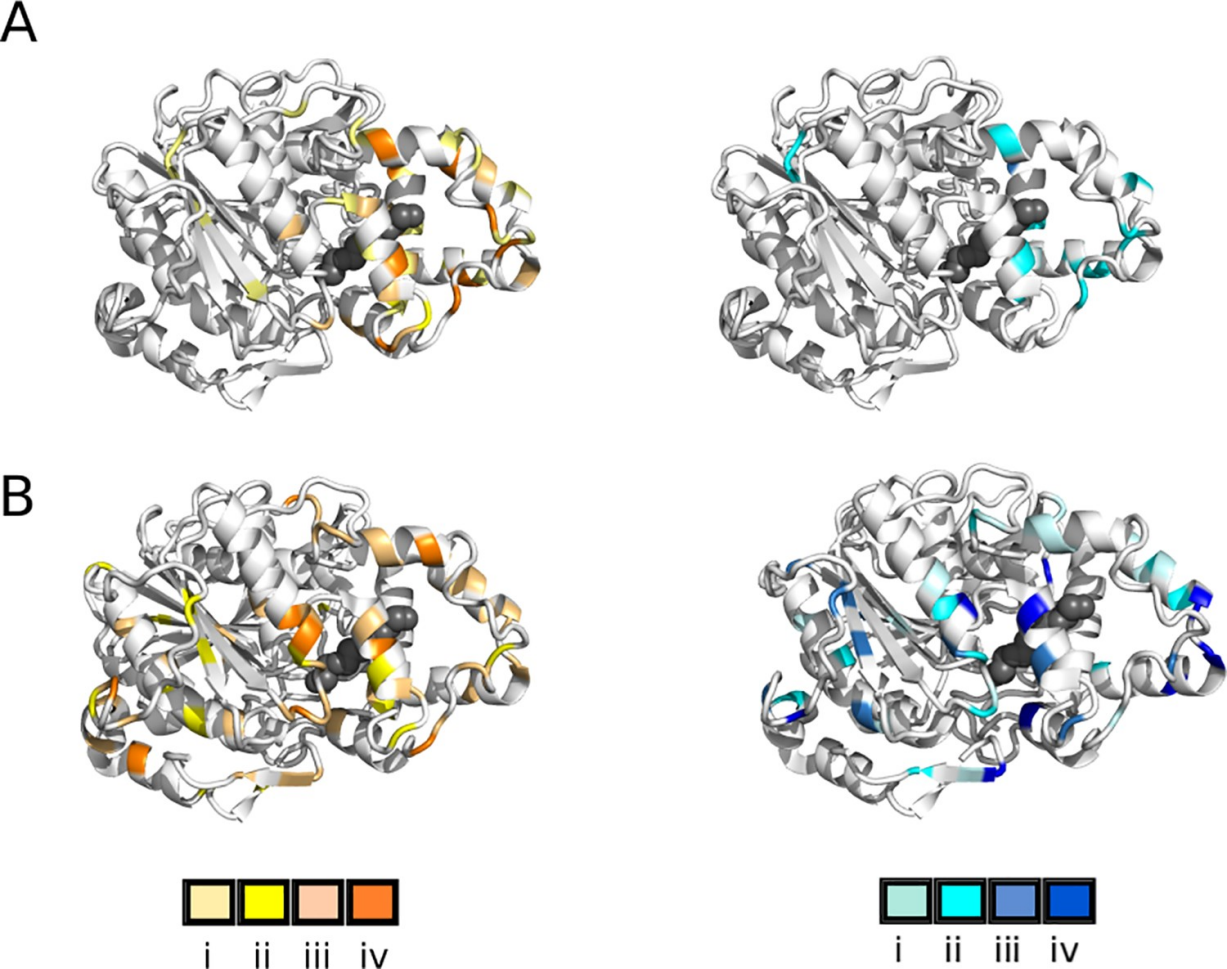
Activity level of the discriminative variants towards short and long chain triglycerides. A: Variants selected from library Random 2. B: Variants selected from libraries Random Tot and Random Rec, combined. Left panels, short-chain activity (hydrolysis of tributyrin): gradient from light yellow (low activity) to orange (high activity). Right panels, long-chain activity (hydrolysis of olive oil): gradient from light (low activity) to dark blue (high activity). Inactive variants coloured as background (gray). Wild-type activity corresponds to shade iii. Where more than one discriminative variant was mutated at the same position, the position is colored according to the variant having the highest activity. A PEG-4 molecule is shown in black spheres, crystallized inside the putative tunnel [22, 29]. Libraries Random Tot and Random Rec are individually represented in S1 Fig. Libraries Random 1 and Random 3 did not yield discriminative variants and are not represented.

Visualizing the positions where substitutions were consistent with discriminative hydrolysis of short-chain triglycerides (tributyrin) or long-chain triglycerides (olive oil) immediately reveal patterns of modified levels of activity (Fig 3). Library Random 2 includes residues linked to phenotypes that confer higher than wild-type activity towards the short-chain substrate but none showed higher than wild-type activity towards the long-chain substrate (Fig 3; S1 Table). In contrast, libraries Random Rec and Random Tot link a greater diversity of residues to high-activity phenotypes, including variants with activity greater than the wild-type, for both short- and long-chain activity (Fig 3; S2 and S3 Tables). The residues that are substituted in high-activity variants are predominantly located in Part 2 (41 residues), while Part 1 contains 12 and Part 3 contains 20 residues associated with high activity, excluding repetitions (S1 Fig).

We noted that the majority of discriminative variants displaying activity greater than wild-type for short- or long-chain hydrolysis and including substitutions in Part 1 or 3 also included at least one substitution in Part 2, with the exception of Gln65Leu, Pro84Gln, Ser359Ile, Ser377Arg, and Ala444Thr. Although the role of individual substitutions in the randomized variants has not been deconvoluted, this suggests that substitutions within Part 2 may be main contributors to the observed discriminative phenotypes. In one specific case, we were able to compare the point-substituted Arg262His variant alone and in conjunction with two additional substitutions. Interestingly, Arg262His (Part 2) alone confered low activity towards long-chain triglycerides (S1 Table), yet when selected in combination with substitutions Leu274Trp and Gly432Asp (Part 3), it confered high activity towards the long-chain substrate (S3 Table). The increased activity is apparently due to an additive or synergistic effect of the combined substitutions.

#### Analysis of discriminative quality of the variants

**Fig 4:**
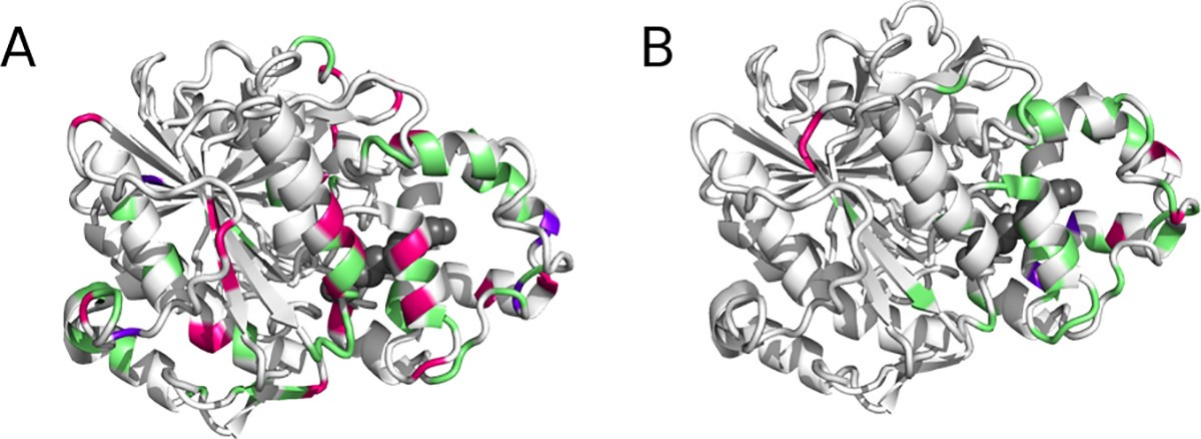
Residues substituted in variants conferring discriminative activity. A) Library Random 2. B: Libraries Random Tot and Random Rec, combined. Green: clear discrimination for hydrolysis of short-chain triglycerides (tributyrin). Magenta: clear discrimination for hydrolysis of the long-chain triglycerides (olive oil). Purple: residues substituted in distinct variants showing opposite discriminative phenotypes. A PEG-4 molecule is shown in black spheres, crystallized inside the putative tunnel [22, 29].

From the perspective of long-chain *vs* short-chain substrate discrimination, clear patterns were also revealed (Fig 4; S1–S4 Tables).

**Short chain discrimination:** Short-chain discrimination was strongly biased to substitutions in Part 2: it is compelling to note that more than half (34) of the total mutations found (55) in the fully randomized libraries (Random Tot and Random Rec) belong to Part 2, and eight among these 34 residues were also identified upon screening library Random 2 (Fig 3, S1 and S2 Tables). Furthermore, all but one of the additional 21 residues identified outside of Part 2 belong to variants including at least one substitution in Part 2. The sole exception, Ser377Arg, was a point-substituted variant offering weak discrimination for short-chain triglycerides (S2 Fig, S2 Table). These results clearly identify this tunnel region as being key for fatty-acid substrate recognition, supporting the hypothesis based on co-crystallized PEG-4 and previous engineering results [22, 29].

A number of the selected, randomized variants held a single substitution, allowing for straightforward analysis of their impact on substrate recognition. Point-mutations that confer strong short-chain discriminative activity are Phe222Ile, Leu225Pro, Gly237Asp, Gly240Cys, and Ala244Val, while point-mutants Leu235Pro and Pro338Glu/Thr confer weak short-chain discrimination (S1–S3 Tables). The helix-loop-helix motif in Part 2, formally delimited by residues Ser217-His245 according to the crystal structure and belonging to the Cal-A lid domain,[29] is thus a hot-spot for creation of short-chain discrimination.

Additional information was obtained from positions that where mutations were observed at a higher frequency, accompanied by different further mutations. This suggests that they are key to the observed property. Residues selected more than once within distinct variants are Phe222Ile (3×), Gly237 substituted either as Asp/Cys/Ser, Ala244 as Thr/Val, Pro338 substituted either as Thr/Gln and Ala342 substituted either as Val (2×)/Asp (S2 and S3 Tables). In addition, the combination of Pro229Leu/Gly237Ser (selected 2×) conferred strong short-chain discrimination. Three further residues appeared twice each among the weak short-chain discriminative pool: Thr221Arg/Lys, Ala236Thr/Val and Asn291Lys/Tyr (S1 Table). All the above residues belong to Part 2 (residues 211–350), confirming the importance of this region in conferring chain-length selectivity.

**Long chain discrimination:** Variants giving rise to long-chain discrimination held mutations that were more scattered throughout the protein: 6 mutations were found in Part 1, 12 in Part 2 and 10 in Part 3 (Fig 4B). Pro84Gln (Part 1), Gly232Cys (Part 2) and Ser359Ile (Part 3) were point-mutated variants, and unequivocally confer long-chain discrimination. Together, substitutions Gln65Leu (Part 1) and Ala444Thr (Part 3) gave long-chain discrimination. All other long-chain discriminative variants containing any substitution in Parts 1 and/or 3 also contain at least one substitution in Part 2. The positions substituted more than once for this phenotype were Arg262His (2×), and Ala402Val/Glu (2×). Overall, fewer substitutions were identified that give rise to long-chain discrimination relative to short-chain, as may be expected according to the ease of excluding large substrates relative to that of including only large substrates.

**Substitutions found both in short- and long-chain discriminative variants:** Also of interest are positions where substitutions occured both in variants that showed selectivity towards short and long chain triglycerides: Lys265Thr or Gln (short-chain or long-chain discrimination, respectively), Tyr136Asn or Phe (short/long-chain, respectively), Leu289Met or Val (short/long-chain, respectively), Ala402Val or Ala/Glu (short/long-chain, respectively) and Arg255His or Ser (short/long-chain, respectively) (Fig 4). Each of the above variants contained further mutations which may be determinants of selectivity. The impact of accompanying mutations is clearly illustrated by Gly232Cys: alone, it sufficed to confer long-chain discrimination, while its combination with Gly227Ser, and Lys265Thr inverted selectivity. Considering that Lys265 was substituted as Gln in a variant with long-chain discrimination (along with substitutions Val238Leu, Arg262His, Thr293Ser andPro325Thr), it is plausible that the inverted selectivity is attributable to Gly227Ser. Finally, Gly228Ser/Val were included in variants conferring short-chain discrimination (each holding a second substitution in Part 2), yet the same Gly228Ser was included in a variant conferring long-chain discrimination, accompanied by 2 substitutions in Part 3.

Globally, screening the diversified libraries against triglyceride substrates led to the unequivocal identification of previously unreported residues that contribute to substrate recognition and discrimination. Point-substituted variants that confer short-chain discrimination were identified at positions 183, 235, 240, 244, 338 and 377, whereas long-chain discriminators were identified at positions 84, 93, 232 and 359. In addition, our results confirm and lend further support to prior reports of key residues. In particular, Thr221 and Phe222 were found to be crucial to modulate selectivity in the hydrolysis of *trans*- and *cis*-fatty acids [22], while Gly237 had previously been identified as important for short-chain discrimination [23]. Residues Thr221/Leu225/Phe233/Gly237 were involved in increased activity towards sterically hindered substrates [37].

### Quantification of hydrolysis using *p*-NO_2_ phenyl esters of different chain lengths

*p*NO_2_-Phenyl-fatty acids are the reference substrates used in industry assessment of lipase reactivity[34]. *p*-NO_2_-Phenyl-ester derivatives of single fatty acids differ importantly from the triglycerides used in the in-plate screening: *p*-NO_2_-phenolate is an activated leaving group, facilitating hydrolytic reactivity; binding to the enzyme necessarily differs since the volume of a triglyceride is nearly 3× that of a *p*NO_2_-phenyl-fatty acid. We had envisaged applying the rapid triglyceride screening method described above to qualitatively identify promising variants for further quantitative *p*NO_2_-phenyl-fatty acid characterization. However, consistent with the reasons mentioned above, we obtained incomplete correspondence between the two assays, as recently observed in a study of *Candida rugosa lipase 1* [38]. Nonetheless, screening with *p*NO_2_-phenyl-substrates allowed us to gather quantitative insights into chain-length discrimination for all variants that showed correspondence. We report results of assays on clarified *E*. *coli* lysates using *p*NO_2_-phenyl-butyrate, -decanoate, -dodecanoate and–palmitate; assays with *p*NO_2_-phenyl-octanoate produced lower quality results due to background hydrolysis. Results show that wild-type Cal-A displays a slight selectivity towards short-chain fatty acids (activity 60% higher with *p*NO_2_-phenyl-butyrate than *p*NO_2_-phenyl-palmitate, S3 Fig).

**Fig 5:**
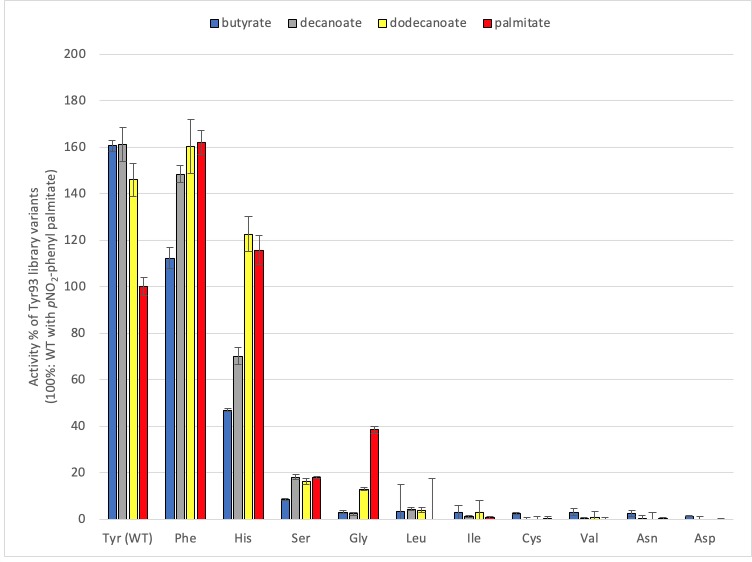
Hydrolytic activity of Tyr93 library variants with *p*-NO_2_-phenyl fatty acids. Assays were performed in triplicate with clarified *E*. *coli* lysates. Activity is reported relative to WT Cal-A with *p*NO_2_-phenyl-palmitate. Its specific activity (S.A) = 0.4 U/mg, is set as 100%.

For variants belonging to the Tyr93 library, activity in clarified lysates is lost when Tyr93 is substituted with Val, Asn, Leu, Cys, Ile and Asp (Fig 5). Surprisingly, selectivity is reversed when Tyr is substituted with the conservative substitutions His and Phe, as well as Gly. Lysate holding the Tyr93Gly variant is 12 times more active toward *p*NO_2_-phenyl palmitate than *p*NO_2_-phenyl butyrate (compared to 2.5 times for Tyr93His), yet its maximum activity with *p*NO_2_-phenyl palmitate is importantly reduced (40% of the wild-type; Fig 5). This illustrates a trade-off between improved discrimination and residual activity, and confirms that activity of variant Tyr93Gly is markedly higher with long-chain substrate–whether a single-chain *p*NO_2_-phenyl derivative or a triglyceride–than with short-chain substrates.

**Fig 6:**
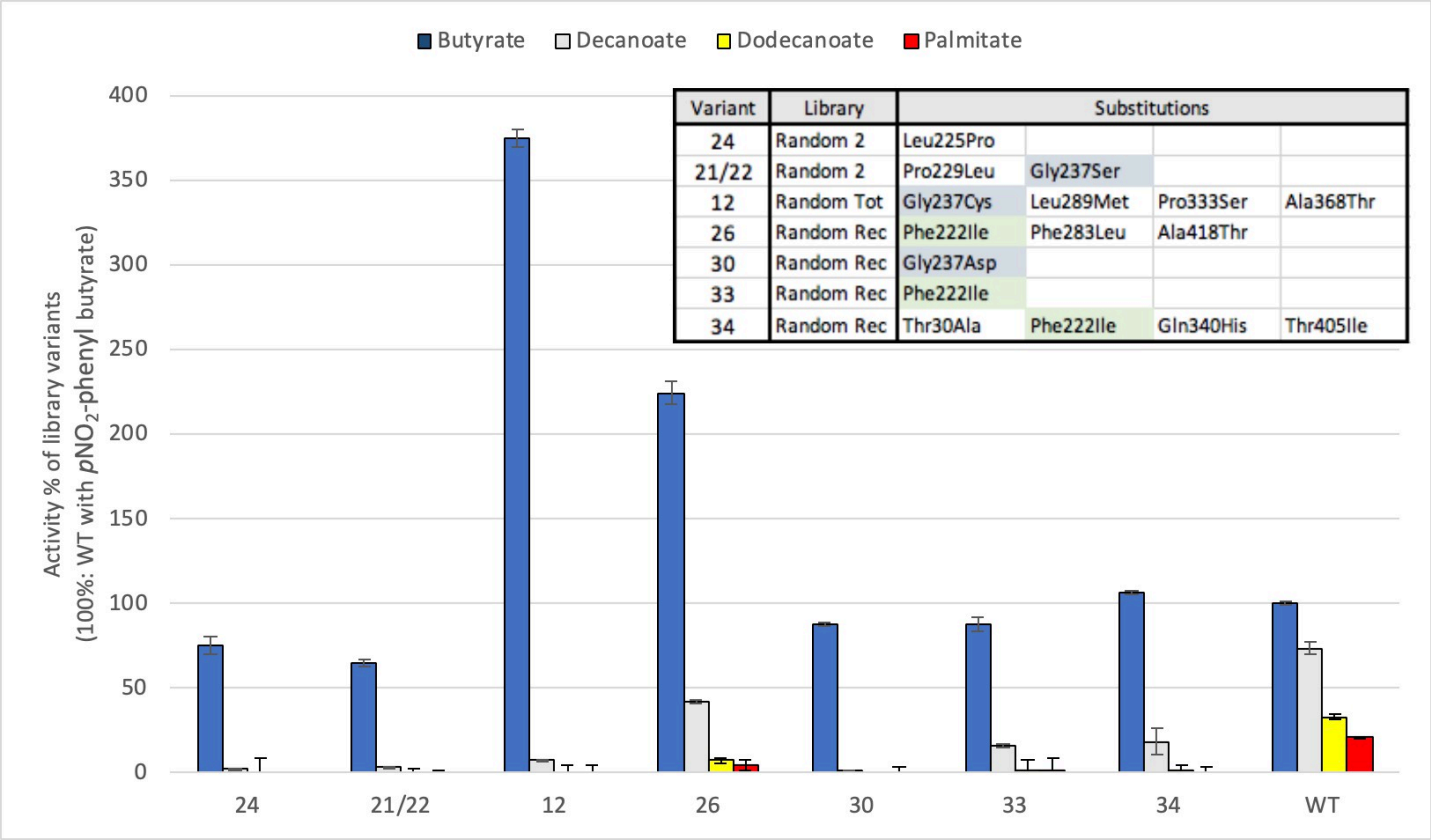
Hydrolytic activity of discriminative variants from randomized libraries with *p*-NO_2_-phenyl fatty acids. Assays were performed in triplicate with clarified *E*. *coli* lysates. Activity is reported relative to WT Cal-A with *p*NO_2_-phenyl-butyrate. Its specific activity (S.A.) = 0.4 U/mg, is set as 100%. Substitutions included in each variant are inset and recurring mutations are highlighted. Variant number is shown as per S1–S3 Tables.

Among the 53 variants from randomized libraries identified as discriminative when screened against triglycerides, 7 showed discrimination in the *p*NO_2_-phenyl-fatty acid assays. We quantified their activity towards these industrially preferred benchmarking compounds (Fig 6). Consistent with their lack of activity on the olive oil solid medium, the variants all show selective hydrolysis of short-chain *p*NO_2_-phenyl-fatty acid substrates. Their activity in the lysate is in the same range as, or greater than, the wild-type towards *p*NO_2_-phenyl-butyrate and they are poorly active or inactive towards all longer chain substrates. We thus demonstrate that these variants exhibiting high activity only toward short-chain triglycerides (solid medium assay) show the same selectivity toward short-chain *p*NO_2_-phenyl-fatty acids.

**Fig 7:**
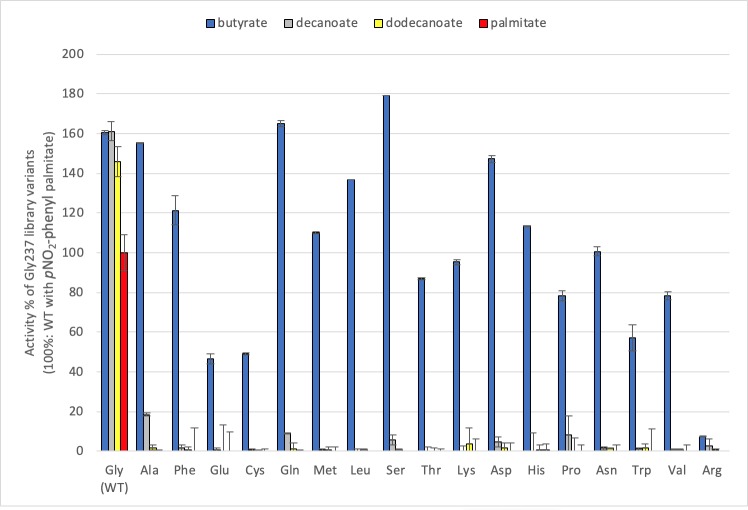
Hydrolytic activity of Gly237 variants with *p*-NO_2_-phenyl fatty acids. Assays were performed in triplicate with clarified *E*. *coli* lysates. Activity is reported relative to WT Cal-A with *p*NO_2_-phenyl-palmitate. Its specific activity (S.A) = 0.4 U/mg, is set as 100%.

Among these variants, substitutions Gly237Asp and Phe222Ile of the 217–245 helix-loop-helix motif appear as point mutations, confirming their role in improved discrimination (S2 Fig). Interestingly, position 237 was previously reported to be important for discrimination of short and long-chain fatty acids according to analysis of 4 different substitutions (Ala, Leu, Tyr, Val) [23]. Here, three distinct discriminative variants carry Gly237 substitutions, as identified in the triglyceride screening (Asp, Cys, Ser) that are an addition to those that were previously characterized [23]. To verify their effect in isolation, we broadened the deconvolution at this position (Fig 7). When Gly237 is substituted with any amino acid, strong short-chain discrimination is established; Cal-A loses its ability to process longer substrates. It thus appears that any steric hindrance at position 237 precludes binding of fatty acid esters longer than butyrate, confirming and extending prior observations of the importance of this residue in chain-length discrimination [23].

## Conclusions

Through the application of our Golden Gate-based strategy for enzyme engineering, we generated focused randomized libraries covering specific parts of Cal-A lipase to scan for variants improved in the discriminative hydrolysis of short- *vs* long-chain triglycerides. This approach allowed introducing the entire mutational load in selected regions, without putting a strain on distal parts of the protein, which is not readly achievable with traditional randomization methods. Furthermore, automated visualization of activity screening results on the structure allowed straightforward identification of a key functional hot-spot.

By those means, we have identified the 217–245 helix-loop-helix region in Part 2 of Cal-A as being fundamental for triglyceride recognition. While it was not possible to dock a triglyceride in Cal-A without assuming extensive conformational changes to fit it in the tunnel [11], our findings confirm that Part 2 is a key to triglyceride recognition. We also identified several previously unreported point substitutions that improve discrimination both for short and long-chain triglycerides. Although there was an incomplete correlation between hydrolysis of triglycerides and *p-*NO_2_-phenyl fatty acids, limited analysis using the latter has provided some quantitative insights on improved selectivity.

## Materials and methods

### Materials, strains, vectors and culture conditions

Unless otherwise stated, DNA primers and chemicals were purchased from Sigma-Aldrich. The rectangular Nunc OmniTray dishes were purchased from Thermo Fisher Scientific. Restriction enzymes were obtained from New England Biolabs, while the TAKARA ligase was from Clontech. Protein markers were purchased from Thermofisher and New England Biolabs. Phusion Green High-Fidelity DNA Polymerase and the PureLink PCR Purification Kit (Invitrogen) were also from Thermo Fisher Scientific. Taq polymerase was purchased from Biobasics. The QuikChange Lightning Site-Directed Mutagenesis Kit and the GeneMorph II Random Mutagenesis Kit from Agilent Technologies were used for mutagenesis. The Monarch Plasmid Miniprep Kit and the Monarch DNA Gel Extraction Kit were purchased from New England Biolabs. A Beckman Coulter Biomek NX^p^ Robot was used to perform the automated triglyceride screening.

Cal-A parts were provided in mother vectors (pM269; chloramphenicol resistant) and codon optimised by ATUM (https://www.atum.bio, formerly DNA2.0 California, USA) as were the linearized daughter vectors (pD441pelB, pD441OmpA; kanamycin resistant). ‘Mother vector’ refers to a plasmid carrying a part, and ‘daughter vector’ is the expression plasmid. Sequencing of the DNA was performed by either the Genomic Platform of IRIC (Institute for Research in Immunology and Cancerology), Université de Montréal, or the Centre d'Innovation Génome Québec at McGill University (QC, Canada).

The CaCl_2_ method was used for the preparation of chemically competent *E*. *coli* BL21 (DE3). Unless otherwise specified, transformed *E*. *coli* strains were cultured on LB agar and in LB broth containing kanamycin (50 μg/mL) and chloramphenicol (35 μg/mL) or ampicillin (100 μg/mL), depending on the resistance marker, overnight (for 16 hours) at 37°C, with shaking at 250 rpm when appropriate.

### Assembly of plasmids and library generation

Restriction digestion of daughter plasmids and wild-type or mutated parts was performed using SapI or BsaI restriction enzymes. Mutant libraries were obtained as described in previous work [11]. Design and assembly *in silico* of all genetic constructs and analysis of the sequencing results were carried out using the SnapGene software (GSL Biotech; available at snapgene.com).

### Screening of Cal-A variants through automation by liquid handling robot

Screening with triglycerides was carried out on tributyrin (short-chain triglyceride) and olive oil (long-chain triglyceride) plates as described previously [11]. A halo of clearance around a colony grown on tributyrin-containing medium indicates activity toward the short-chain substrate while a halo of fluorescence around a colony on olive oil/rhodamine-containing medium indicates activity toward long-chain substrate. Wild-type Cal-A activity was assigned a value of 3 and haloes of similar size and intensity (either degree of clearance for tributyrin, or fluorescence intensity for olive oil/rhodamine) upon visual inspection of triplicates were assigned that value. Larger and more intense haloes were clearly distinguished and were assigned the maximal value of 4. Less active variants generated smaller and less intense haloes that assigned the value of 2 or 1 (smallest/faintest haloes).

### Computational method for phenotypic characterization

To facilitate the establishment and analysis of genotype/phenotype relationships in our dataset, a Python script was developed. DNA sequencing results were classified by phenotype according to the results of screening with triglycerides. One phenotype qualified the level of activity observed toward a given triglyceride substrate (halo formation). The second phenotype identified short- or long-chain discrimination based on the relative activity with each of the triglyceride substrates; mutated residues that contributed to both phenotypes were assigned to a third discriminative category.

To simplify 3D analysis, each phenotype was assigned a numerical value that was used to modify the B-factor column in 2VEO pdb file using the data2bfactor.py script (http://pldserver1.biochem.queensu.ca/~rlc/work/pymol/data2bfactor.py) in the molecular graphics program PyMOL. Coloring by B-factor number revealed the localization of the residues contributing to each phenotype. The script is available upon request.

### Screening Cal-A variants with *p*-NO_2_-phenyl esters

For the liquid screening using *p*-NO_2_-phenyl esters, the variants and the negative control (*E*. *coli* BL21(DE3) transformed with pD441pelB that does not contain the Cal-A gene) were expressed in 10 mL ZYP-5052 auto-inducing medium, described by Studier [39], supplied with kanamycin, at 30°C over 24 hours. An overnight culture in LB grown at 37°C was used as inoculum (100 μL inoculum for 10 mL of culture). Conditions were determined by a Design of Experiment performed considering different temperatures, volumes and inoculum conditions [11]. The cultures were pelleted by microcentrifuging at 3000 rpm over 10 min and lysed by resuspending in 1 mL of 50 mM Tris, pH 8.0, sonicating until clear and microcentrifuging at 14000 rpm for 10 min. The resulting supernatant was assayed. Spectrophotometric activity measurements were based on the substrate-dependent absorbance change of *p*-NO_2_-phenyl esters of different chain lengths at 405 nm. Assays were routinely done in 200 μL at 45°C, with measurement over 30 min using a *BMG FLUOstar* 96-well plate spectrophotometer.

Unless otherwise stated, the reaction mixture contained *p*-NO_2_-phenyl-fatty acid (0.5 mM), 1% Triton x 100, 50 mM Tris, pH 8.0. A Bradford assay was performed on the lysates. One unit of Cal-A corresponded to the amount of enzyme required to produce 1 μmol of *p*-NO_2_-phenolate per min at 45°C.

The specific activity was calculated according to the following formula:

$$S.A.(\frac{U}{mg})=\frac{\Delta 450}{l\times \varepsilon pNO2phenolate}\times \frac{Vt}{Ve}\times \frac{1}{\left[Ce\right]}$$

where: Δ450 = absorbance slope (min^-1^); l = cuvette length (cm); ε *p*-NO_2_-phenolate = 18.1 mM^-1^ cm^-1^; Vt = total reaction volume in mL; Ve = total enzyme volume in mL; [Ce] = concentration of total protein in lysate in mg/mL.

## Supporting information

S1 Table Activity for short-chain and long-chain discriminative Cal-A variants selected from library Random 2 during screening against triglyceride substrates. (DOCX)

S2 Table Activity for discriminative variants selected from library Random Rec during screening against triglyceride substrates. (DOCX)

S3 Table Activity for discriminative variants selected from library Random Tot during screening against triglyceride substrates. (DOCX)

S4 Table Residues that appear in both short-chain and long-chain discriminative variants. (DOCX)

S1 Fig Activity for discriminative variants selected from libraries Random Tot and Random Rec towards short- and long-chain triglycerides. (DOCX)

S2 Fig Identification of key residues belonging to discriminative variants, classified according to their discriminative nature. (DOCX)

S3 Fig Hydrolytic activity of wild-type Cal-A with *p*-NO_2_-phenyl fatty acids. (DOCX)
