# Supplementary material for: Holistic engineering of Cal-A lipase chain-length selectivity identifies triglyceride binding hot-spot
Source: PLoS One. 2019 Jan 14;14(1):e0210100. doi: 10.1371/journal.pone.0210100 (PMC6331120; doi:10.1371/journal.pone.0210100)
Supplement: S1 Table — (DOCX) [file pone.0210100.s001.docx]

**S1 Table. Activity for short-chain and long-chain discriminative Cal-A variants selected from library Random 2 during screening against triglyceride substrates.**

Hydrolytic activity of variants towards the short-chain triglyceride tributyrin and the long-chain substrate olive oil was categorized as very low (1), low (2), medium (3) or high (4). The value (0) indicates no activity detected towards that substrate. Wild-type Cal-A activity value is 3. The color code is identical to Fig 3. Twenty-one discriminative variants were identified in this library: eighteen variants showing short-chain discrimination and three variants showing long-chain discrimination.

1. Random 2 library variants that discriminate for short-chain fatty acids

| Variant | Activity | | Residue |  |  | |
| --- | --- | --- | --- | --- | --- | --- |
|  | Short-chain | Long-chain |  | **WT** | **Mut** |  |
| 101 | 1 | 0 | 217 | S | I |  |
| 101 | 1 | 0 | 244 | A | T |  |
| 101 | 1 | 0 | 315 | A | T |  |
| 106 | 1 | 0 | 236 | A | V |  |
| 106 | 1 | 0 | 331 | A | T |  |
| 110 | 1 | 0 | 243 | L | P |  |
| 110 | 1 | 0 | 300 | P | S |  |
| 110 | 1 | 0 | 327 | F | L |  |
| 111 | 1 | 0 | 236 | A | T |  |
| 111 | 1 | 0 | 298 | E | K |  |
| 111 | 1 | 0 | 307 | Q | H |  |
| 111 | 1 | 0 | 319 | V | E |  |
| 112 | 2 | 0 | 227 | G | S |  |
| 112 | 2 | 0 | 232 | G | C |  |
| 112 | 2 | 0 | 265 | K | T |  |
| 114 | 1 | 0 | 221 | T | R |  |
| 114 | 1 | 0 | 291 | N | Y |  |
| 117 | 3 | 0 | 225 | L | P |  |
| 118 | 1 | 0 | 221 | T | K |  |
| 118 | 1 | 0 | 234 | A | T |  |
| 118 | 1 | 0 | 271 | G | S |  |
| 118 | 1 | 0 | 291 | N | K |  |
| 121 | 4 | 0 | 229 | P | L |  |
| 121 | 4 | 0 | 237 | G | S |  |
| 122 | 4 | 0 | 229 | P | L |  |
| 122 | 4 | 0 | 237 | G | S |  |
| 124 | 3 | 0 | 228 | G | S |  |
| 124 | 3 | 0 | 258 | A | S |  |
| 126 | 4 | 3 | 244 | A | V |  |
| 127 | 4 | 0 | 248 | M | L |  |
| 127 | 4 | 0 | 294 | N | I |  |
| 130 | 4 | 2 | 223 | T | S |  |
| 130 | 4 | 2 | 244 | A | V |  |
| 130 | 4 | 2 | 263 | T | A |  |
| 130 | 4 | 2 | 285 | N | I |  |
| 130 | 4 | 2 | 290 | V | D |  |
| 130 | 4 | 2 | 306 | K | N |  |
| 131 | 1 | 0 | 228 | G | V |  |
| 131 | 1 | 0 | 231 | A | T |  |
| 133 | 3 | 1 | 235 | L | P |  |
| 135 | 3 | 0 | 237 | G | C |  |
| 135 | 3 | 0 | 251 | F | Y |  |
| 135 | 3 | 0 | 275 | P | Q |  |
| 135 | 3 | 0 | 335 | E | D |  |
| 135 | 3 | 0 | 342 | A | V |  |
| 136 | 3 | 0 | 218 | A | V |  |
| 136 | 3 | 0 | 240 | G | V |  |

1. Random 2 library variants that discriminate for long-chain fatty acids

| Variant | Activity | | Residue |  |  |
| --- | --- | --- | --- | --- | --- |
|  | **Short-chain** | **Long-chain** |  | **WT** | **Mut** |
| 116 | 1 | 2 | 238 | V | L |
| 116 | 1 | 2 | 262 | R | H |
| 116 | 1 | 2 | 265 | K | Q |
| 116 | 1 | 2 | 293 | T | S |
| 116 | 1 | 2 | 325 | P | T |
| 132 | 1 | 2 | 232 | G | C |
| 134 | 1 | 2 | 254 | A | E |
| 134 | 1 | 2 | 324 | F | C |
