# Supplementary material for: Holistic engineering of Cal-A lipase chain-length selectivity identifies triglyceride binding hot-spot
Source: PLoS One. 2019 Jan 14;14(1):e0210100. doi: 10.1371/journal.pone.0210100 (PMC6331120; doi:10.1371/journal.pone.0210100)
Supplement: S2 Table — (DOCX) [file pone.0210100.s002.docx]

**S2 Table. Activity for discriminative variants selected from library Random Rec during screening against triglyceride substrates.**

Hydrolytic activity of variants towards the short-chain triglyceride tributyrin and the long-chain substrate olive oil was categorized as very low (1), low (2), medium (3) or high (4). The value (0) indicates no activity detected towards that substrate. Wild-type Cal-A activity value is 3. The color code is identical to Fig 3. Eighteen discriminative variants were identified in this library: twelve variants showing short-chain discrimination and six variants showing long-chain discrimination.

1. Random Rec library variants that discriminate for short-chain fatty acids

| Variant | Activity |  | Residue |  |  |
| --- | --- | --- | --- | --- | --- |
|  | Short-chain | Long-chain |  | WT | Mut |
| 22 | 4 | 0 | 302 | A | S |
| 22 | 4 | 0 | 334 | D | Y |
| 22 | 4 | 0 | 342 | A | E |
| 23 | 3 | 2 | 250 | S | F |
| 23 | 3 | 2 | 335 | E | V |
| 23 | 3 | 2 | 342 | A | V |
| 23 | 3 | 2 | 412 | V | I |
| 24 | 3 | 2 | 312 | Q | K |
| 24 | 3 | 2 | 435 | F | L |
| 26 | 4 | 0 | 222 | F | I |
| 26 | 4 | 0 | 283 | F | L |
| 26 | 4 | 0 | 418 | A | T |
| 30 | 4 | 0 | 237 | G | D |
| 32 | 3 | 1 | 247 | D | G |
| 32 | 3 | 1 | 249 | E | G |
| 32 | 3 | 1 | 305 | L | V |
| 33 | 4 | 0 | 222 | F | I |
| 34 | 4 | 0 | 30 | T | A |
| 34 | 4 | 0 | 222 | F | I |
| 34 | 4 | 0 | 340 | Q | H |
| 34 | 4 | 0 | 405 | T | I |
| 37 | 3 | 0 | 233 | F | I |
| 37 | 3 | 0 | 287 | F | L |
| 39 | 3 | 2 | 338 | P | Q |
| 42 | 3 | 2 | 377 | S | R |
| 45 | 3 | 2 | 338 | P | T |

1. Random Rec library variants that discriminate for long-chain fatty acids

| Variant | Activity | | Residue |  |  |
| --- | --- | --- | --- | --- | --- |
|  | Short-chain | Long-chain |  | WT | Mut |
| 21 | 2 | 3 | 84 | P | Q |
| 27 | 2 | 3 | 359 | S | I |
| 28 | 3 | 4 | 24 | T | I |
| 28 | 3 | 4 | 220 | D | N |
| 28 | 3 | 4 | 341 | P | S |
| 28 | 3 | 4 | 402 | A | E |
| 29 | 2 | 3 | 228 | G | S |
| 29 | 2 | 3 | 402 | A | V |
| 29 | 2 | 3 | 407 | P | A |
| 31 | 0 | 1 | 65 | Q | L |
| 31 | 0 | 1 | 444* | A | T |
| 44 | 3 | 4 | 255 | R | S |
| 44 | 3 | 4 | 257 | N | Y |

*Residue 444 is not represented in the 3D structure because it is not resolved in the original 2VEO PDB file (far C-term).
