# Supplementary material for: Holistic engineering of Cal-A lipase chain-length selectivity identifies triglyceride binding hot-spot
Source: PLoS One. 2019 Jan 14;14(1):e0210100. doi: 10.1371/journal.pone.0210100 (PMC6331120; doi:10.1371/journal.pone.0210100)
Supplement: S3 Table — (DOCX) [file pone.0210100.s003.docx]

**S3 Table. Activity for discriminative variants selected from library Random Tot during screening against triglyceride substrates.**

Hydrolytic activity of variants towards the short-chain triglyceride tributyrin and the long-chain substrate olive oil was categorized as very low (1), low (2), medium (3) or high (4). The value (0) indicates no activity detected towards that substrate. Wild-type Cal-A activity value is 3. The color code is identical to Fig 3. Fourteen discriminative variants were identified in this library: ten variants showing short-chain discrimination and four variants showing long-chain discrimination.

1. Random Tot library variants that discriminate for short-chain fatty acids

| Variant | Activity | | Residue |  |  | |  |
| --- | --- | --- | --- | --- | --- | --- | --- |
|  | Short-chain | Long-chain |  | WT | | Mut | |
| 3 | 3 | 0 | 240 | G | | C | |
| 5 | 3 | 1 | 306 | K | | R | |
| 5 | 3 | 1 | 336 | I | | F | |
| 5 | 3 | 1 | 382 | K | | N | |
| 5 | 3 | 1 | 433 | K | | R | |
| 6 | 3 | 1 | 181 | E | | G | |
| 6 | 3 | 1 | 313 | A | | T | |
| 6 | 3 | 1 | 402 | A | | V | |
| 9 | 2 | 0 | 128 | G | | D | |
| 9 | 2 | 0 | 136 | Y | | N | |
| 9 | 2 | 0 | 144 | G | | S | |
| 9 | 2 | 0 | 255 | R | | H | |
| 10 | 2 | 0 | 232 | G | | C | |
| 10 | 2 | 0 | 428 | Q | | L | |
| 12 | 3 | 0 | 237 | G | | C | |
| 12 | 3 | 0 | 289 | L | | M | |
| 12 | 3 | 0 | 333 | P | | S | |
| 12 | 3 | 0 | 369 | A | | T | |
| 13 | 3 | 2 | 253 | E | | G | |
| 13 | 3 | 2 | 268 | R | | S | |
| 14 | 2 | 0 | 290 | V | | D | |
| 14 | 2 | 0 | 424 | L | | M | |
| 17 | 3 | 1 | 29 | G | | V | |
| 17 | 3 | 1 | 41 | K | | M | |
| 17 | 3 | 1 | 211 | H | | Q | |
| 17 | 3 | 1 | 251 | F | | Y | |
| 17 | 3 | 1 | 284 | L | | S | |
| 18 | 3 | 0 | 190 | T | | I | |
| 18 | 3 | 0 | 226 | N | | S | |
| 18 | 3 | 0 | 406 | T | | S | |
| 18 | 3 | 0 | 410 | D | | E | |

1. Random Tot library variants that discriminate for long-chain fatty acids

| Variant | Activity | | Residue |  |  |
| --- | --- | --- | --- | --- | --- |
|  | Short-chain | Long-chain |  | WT | Mut |
| 4 | 0 | 1 | 22 | Y | C |
| 4 | 0 | 1 | 27 | N | S |
| 4 | 0 | 1 | 307 | Q | R |
| 4 | 0 | 1 | 344 | T | A |
| 4 | 0 | 1 | 371 | I | T |
| 11 | 2 | 3 | 136 | Y | F |
| 11 | 2 | 3 | 289 | L | V |
| 11 | 2 | 3 | 357 | N | D |
| 15 | 3 | 4 | 262 | R | H |
| 15 | 3 | 4 | 274 | L | W |
| 15 | 3 | 4 | 432 | G | D |
| 20 | 2 | 3 | 224 | F | Y |
| 20 | 2 | 3 | 339 | Y | H |
| 20 | 2 | 3 | 372 | F | L |
| 20 | 2 | 3 | 396 | T | I |
