# Supplementary material for: Holistic engineering of Cal-A lipase chain-length selectivity identifies triglyceride binding hot-spot
Source: PLoS One. 2019 Jan 14;14(1):e0210100. doi: 10.1371/journal.pone.0210100 (PMC6331120; doi:10.1371/journal.pone.0210100)
Supplement: S4 Table — (DOCX) [file pone.0210100.s004.docx]

**S4 Table.** Residues that appear in both short-chain and long-chain discriminative variants.

1. Random 2 library:

| Residue | WT | Mut | Variant |
| --- | --- | --- | --- |
| Short-chain discrimination |  |  |  |
| 232 | G | C | 112 |
| 265 | K | T | 112 |
| Long-chain discrimination | | |  |
| 232 | G | C | 132 |
| 265 | K | Q | 116 |

1. Random Rec and Random Tot libraries:

| Residue | WT | Mut | | Variant | |
| --- | --- | --- | --- | --- | --- |
| Short-chain discrimination |  |  | |  | |
| 136 | Y | N | | 9 | |
| 289 | L | M | | 12 | |
| 402 | A | V | | 6 | |
| 255 | R | H | | 9 | |
| Long-chain discrimination | | |  | |  |
| 136 | Y | F | | 11 | |
| 289 | L | V | | 11 | |
| 402 | A | E | | 28 | |
| 402 | A | V | | 29 | |
| 255 | R | S | | 44 | |
