# Supplementary material for: Holistic engineering of Cal-A lipase chain-length selectivity identifies triglyceride binding hot-spot
Source: PLoS One. 2019 Jan 14;14(1):e0210100. doi: 10.1371/journal.pone.0210100 (PMC6331120; doi:10.1371/journal.pone.0210100)
Supplement: S1 Fig — (DOCX) [file pone.0210100.s005.docx]

**S1 Fig. Activity for discriminative variants selected from libraries Random Tot and Random Rec towards short- and long-chain triglycerides.**

**
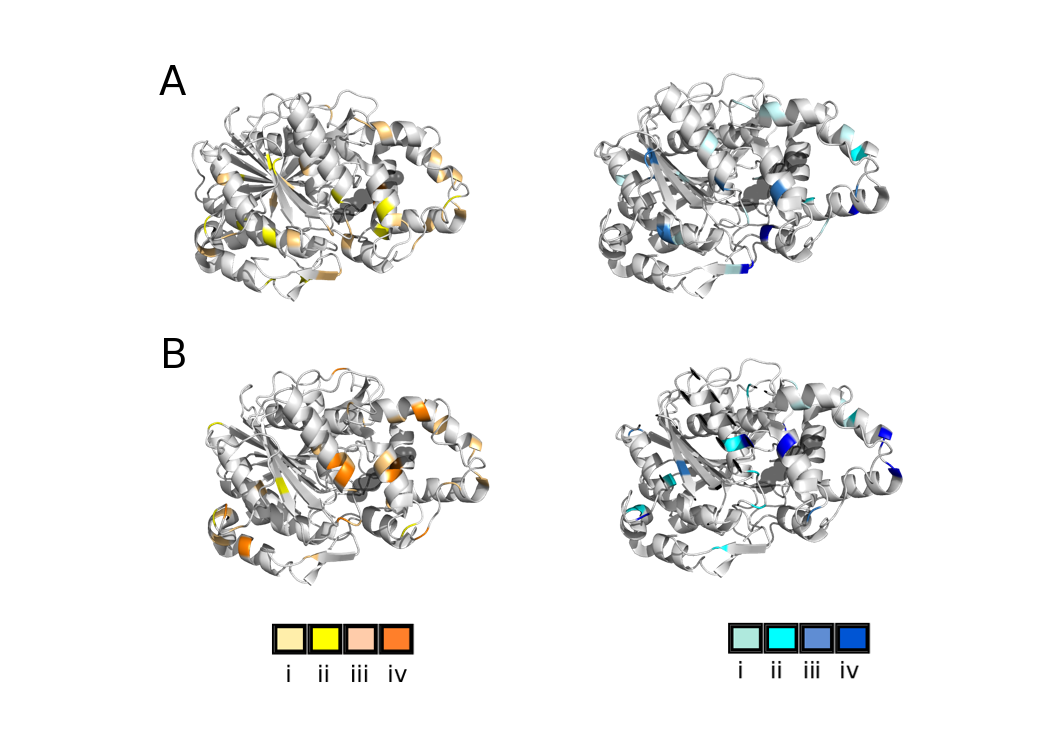
**

The residues that were substituted in the selected, discriminative variants are colored according to the activity of the variant towards each substrate. A: Discriminative variants selected from library Random Tot. B: Discriminative variants selected from library Random Rec. Left panels, short-chain activity (hydrolysis of tributyrin): gradient from light yellow (low activity) to orange (high activity). Right panels, long-chain activity (hydrolysis of olive oil): gradient from light to dark blue. Wild-type level activity corresponds to shade iii. Where no activity was detected towards a substrate, no color was assigned. Where more than one variant was mutated at the same position, the position is colored according to the variant having the highest activity. A PEG molecule is shown in black spheres, crystallized inside the putative tunnel (1).

**Supplemental Reference:**

1. Ericsson DJ, Kasrayan A, Johansson P. X-ray structure of *Candida antarctica* lipase A shows a novel lid structure and a likely mode of interfacial activation. *J Mol. Biol*. 2008; 375: 109-19. doi: 10.1016/j.jmb.2007.10.079. PubMed PMID: 2919708792473828267related:q7v8bq3jhCgJ.
