# Supplementary material for: Holistic engineering of Cal-A lipase chain-length selectivity identifies triglyceride binding hot-spot
Source: PLoS One. 2019 Jan 14;14(1):e0210100. doi: 10.1371/journal.pone.0210100 (PMC6331120; doi:10.1371/journal.pone.0210100)
Supplement: S2 Fig — (DOCX) [file pone.0210100.s006.docx]

**S2
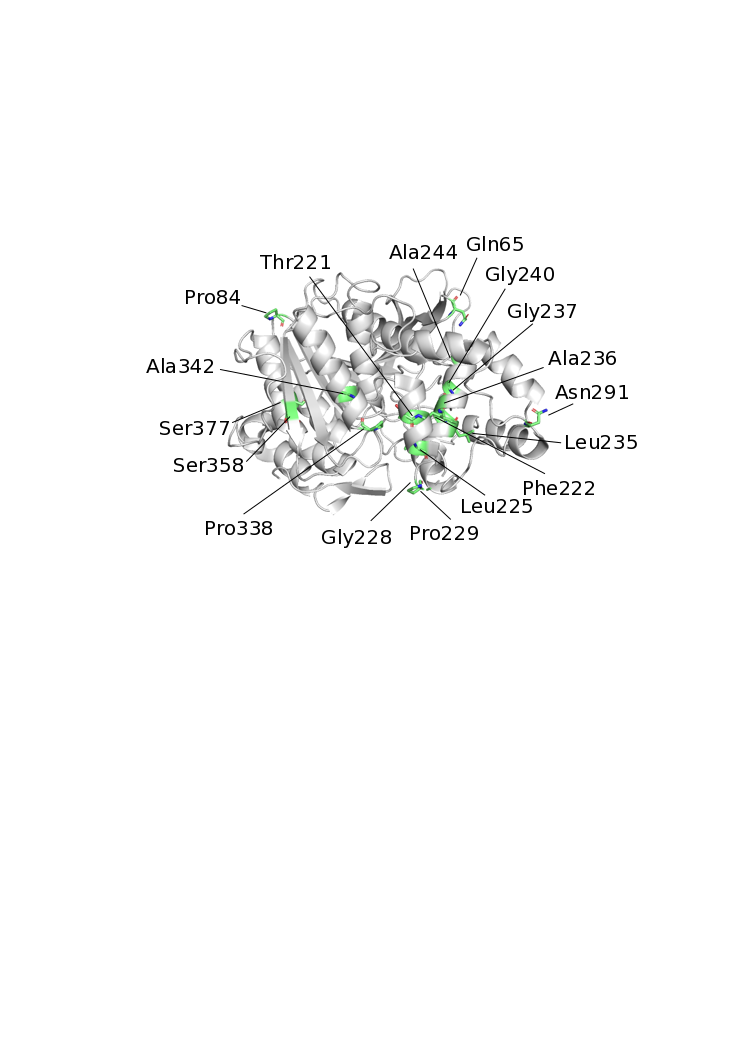
 Fig. Identification of key residues belonging to discriminative variants, classified according to their discriminative nature.**

­­­


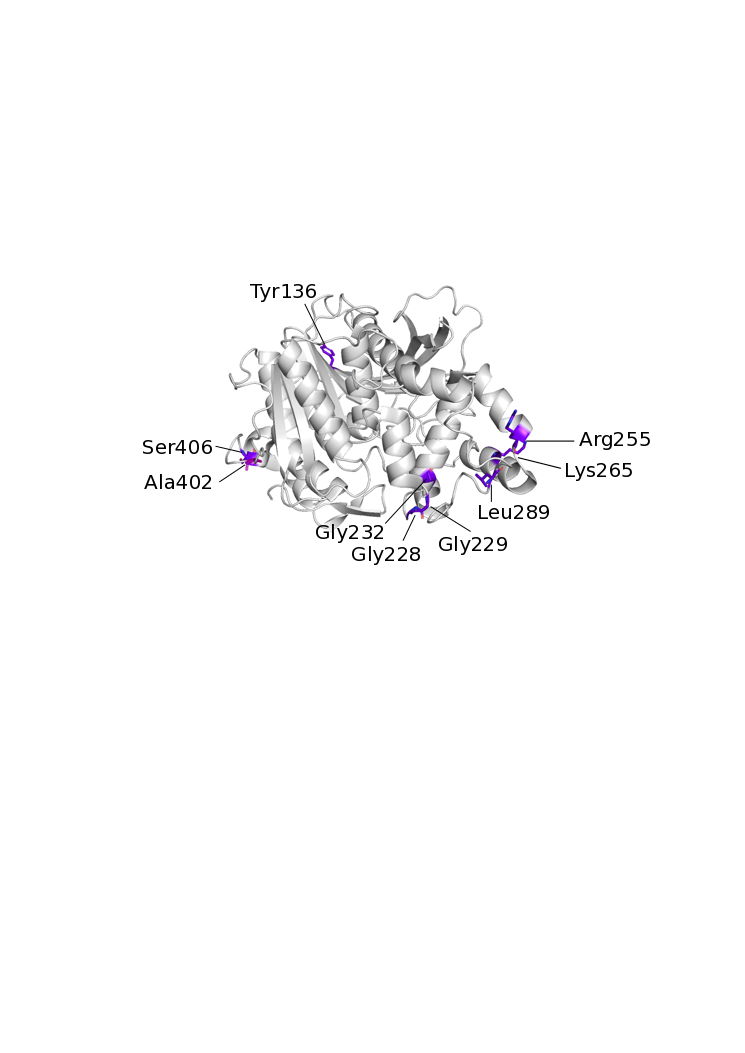

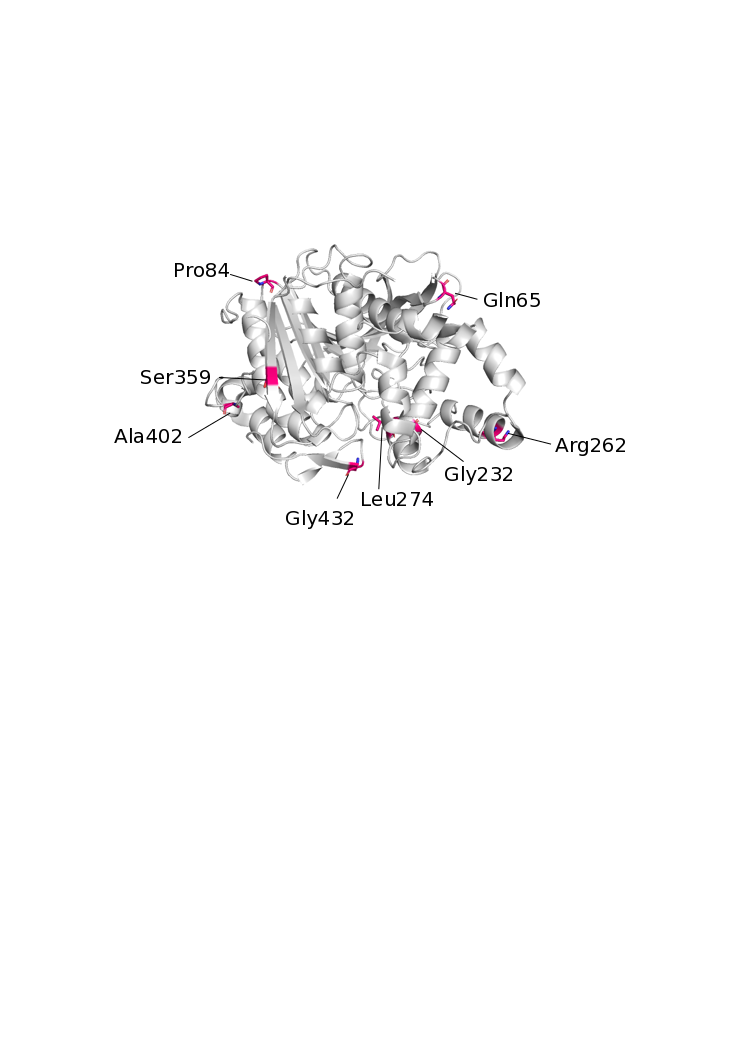


**­­­­­**

­­­

Residues highlighted in green were substituted in randomized variants that discriminate towards short-chain fatty acids, in magenta towards long-chain fatty acids and, in purple, residues that were substituted both in variants that discriminate for short-chain or long-chain fatty acids.
