# Supplementary material for: Holistic engineering of Cal-A lipase chain-length selectivity identifies triglyceride binding hot-spot
Source: PLoS One. 2019 Jan 14;14(1):e0210100. doi: 10.1371/journal.pone.0210100 (PMC6331120; doi:10.1371/journal.pone.0210100)
Supplement: S3 Fig — (DOCX) [file pone.0210100.s007.docx]

**S3 Fig. Hydrolytic activity of wild-type Cal-A with *p*-NO_2_-phenyl fatty acids.**

Assays were performed in triplicate with clarified *E. coli* lysates. Activity is normalized to that of wild-type Cal-A with *p*NO_2_-phenyl-palmitate (= 1, S.A. = 0.4 U/mg).
